# Precision Versus Practicality: A Comprehensive Analysis of Robotic Right Colectomy Versus Laparoscopic Right Colectomy, Future Directions, Biases, Research Gaps, and Their Implications

**DOI:** 10.7759/cureus.52904

**Published:** 2024-01-25

**Authors:** Konstantinos Kossenas, Ioannis Karamatzanis, Olga Moutzouri, Beatrice Catalli, Andreas I Biris, Dimitra Dimaki, Ifigeneia Kokkofiti, Filippos Georgopoulos

**Affiliations:** 1 Surgery, University of Nicosia Medical School, Nicosia, CYP; 2 Internal Medicine, Basildon University Hospital, Basildon, GBR; 3 Medicine, University of Nicosia Medical School, Nicosia, CYP; 4 Pediatrics, Bambino Gesù Children's Hospital, Rome, ITA; 5 Medicine, NHS Scotland, Edinburgh, GBR; 6 Gastroenterology, Al Zahra Hospital, Dubai, ARE

**Keywords:** surgical outcomes, laparoscopic surgery, robotic surgery, colectomy, colorectal cancer

## Abstract

Colorectal cancer is the third most commonly diagnosed cancer in the world and second in cancer-related mortality. It is most prevalent in the developed world and is often associated with lifestyle factors along with age and genetics. The inclusion criteria comprised high-level evidence, such as randomized clinical trials, meta-analyses, and systematic reviews, conducted between 2012 and 2023, that directly compared the two approaches. The review reveals mixed outcomes between robotic right colectomy (RRC) and laparoscopic right colectomy (LRC). The robotic approach was associated with longer operative duration and higher costs but with decreased blood loss and quicker recovery compared to laparoscopy. On the other hand, no major differences were observed regarding lymph node retrieval, duration of hospitalization, and surgical complications. Regarding future directions, it is evident that the focus needs to shift beyond the operative parameters and to patient-centered outcomes, which are underreported. Also, more randomized clinical trials are required, focusing on safety, efficacy, and long-term quality of life. Costs-benefit analyses are required to weigh the benefits of robotic surgery against the implementation and practice costs. Additionally, improvements in surgeons' training may be necessary to reduce the operative duration and potentially decrease operational costs. Finally, standardization of research protocols may be necessary to reduce biases.

## Introduction and background

Colorectal cancer is the third most commonly diagnosed cancer and second in cancer-related mortality [1]. It is most prevalent in developed countries, and its incidence may be attributed to risk factors such as age, genetic predispositions, and lifestyle factors like a diet high in processed red meat, smoking, and long-standing inflammatory bowel disease [2]. Symptoms can be vague and vary among patients but most commonly include changes in bowel habits, blood or bleeding per rectum, and cancer-related symptoms, such as unexplained weight loss [3]. Minimally invasive surgical techniques include laparoscopy and robotic surgery. Within the past decade and with technological advances, laparoscopic surgery has replaced open surgery for the treatment of several different gastrointestinal (GI) malignancies, including upper GI malignancies, hepatic-pancreatic-biliary, and colorectal malignancies too [4,5]. In recent years, robotic surgery has gained momentum and has been implicated in the treatment of colorectal cancer with great results. However, the evidence is still conflicting as to whether robotic surgery outperforms laparoscopic surgery for the treatment of right-sided colorectal cancers [6]. This review aims to perform a comprehensive review of all randomized clinical trials, systematic reviews, and meta-analyses comparing the two approaches, underline the research gaps, outline biases present in the current literature, and suggest future directions for both healthcare and policymakers to implement robotic surgery as efficiently as possible into daily practice while taking into account the patient outcomes. The right-side colorectal cancer will be the main focus of cancers as they are underrepresented in the literature compared to left-sided ones.

## Review

Literature review

A randomized clinical trial by Park et al., in 2012, aimed to investigate the differences between robotic right colectomy (RRC) and laparoscopic right colectomy (LRC). The study included 70 patients in total, separated into two equal groups. Regarding the operative time, the robotic cohort had a notably longer operative duration (195 minutes) when compared to the laparoscopic one (130 minutes) (*P* < 0.001). Also, the overall hospital costs were significantly higher for RRC when compared to LRC (US$12,235 vs. US$10,320 for LRC) (*P* = 0.013). Interestingly, no differences were observed between the two surgical techniques regarding the number of harvested lymph nodes, duration of hospitalization, surgical complications, postoperative pain scores, resection margins, and conversion to open surgery [7].

Xu et al., 2014, performed a meta-analysis to investigate the differences between RRC and LRC. The study concluded that the subjects in the RRC cohorts had significantly less blood loss (*P *= 0.0002), faster bowel function recovery (*P *< 0.00001), and a lower rate of overall postoperative complications (*P *= 0.02). However, RRC required significantly longer operative time (*P *< 0.00001). On the other hand, no significant differences were observed between the two surgical approaches regarding the length of hospitalization, conversion to open surgery, postoperative rate of ileus, anastomotic leakage, and bleeding [8].

In 2015, Chang et al. performed a meta-analysis to compare RRC with LRC. In total, the study included 125,989 patients. The robotic approach led to a shorter duration of hospitalization when compared to LRC, with a mean difference (MD) of -0.65 days (95% confidence interval [CI] -1.02 to -0.27; *P *= 0.0008). Moreover, it exhibited a 22% lower odds ratio (OR) for complication rates (0.78; 95% CI 0.72-0.85; *P* < 0.00001), faster recovery of bowel function (MD -0.58; 95% CI -0.96 to -0.20; *P* = 0.003), less estimated blood loss (MD -19.24; 95% CI -29.38 to -9.09; *P* = 0.0002), and a lower rate of conversion to open surgery (OR 0.56; 95% CI 0.44-0.72; *P* < 0.00001) when compared to LRC. No statistically significant differences were observed regarding the number of harvested lymph nodes. On the other hand, RRC required longer operative time when compared to LRC (MD of 49.25 minutes; 95% CI 36.78-61.72; *P* < 0.00001) [9].

A meta-analysis by Rondelli et al., 2015, aimed to investigate the differences between RRC and LRC. The study included a total of 616 patients. The patients in the RRC cohorts experienced lower blood loss and faster bowel function recovery. No statistically significant differences were observed regarding postoperative outcomes. On the other hand, the robotic approach was associated with longer operative duration and increased costs [10].

Furthermore, Duan et al., in 2016, aimed to compare robotic colectomy (RC) with laparoscopic colectomy (LC), in a large pool of 125,098 patients. The study concluded that RC required more operative time (*P *< 0.01) but had lower blood loss (*P* < 0.01), reduced conversion to open surgery (*P* < 0.01), shorter duration of hospitalization (*P* < 0.01), lower overall postoperative complication rates (*P* < 0.01), and faster bowel function recovery (*P* < 0.01). Finally, no significant differences were observed in the number of lymph nodes harvested (*P* > 0.05) [11].

In a systematic review and meta-analysis, Solaini et al. aimed to compare RRC and LRC in 8,257 patients. The study concluded that LRC had a shorter operative time (standard mean difference [SMD] -0.99; 95% CI -1.4 to -0.6; *P* < 0.001), higher conversion rate to open surgery (relative risk [RR] 1.7; 95% CI 1.1-2.6; *P* = 0.02), and longer time for bowel recovery (SMD 0.85 days; 95% CI 0.16-1.54; *P* = 0.016) when compared to RRC. Moreover, RRC was associated with higher costs (SMD -0.52; 95% CI -0.52 to -0.04; *P* = 0.035). Finally, no major differences were observed between the two techniques for mortality rates (RR 0.47; 95% CI 0.18-1.23; *P* = 0.124) and overall postoperative complications (RR 1.05; 95% CI 0.9-1.2; *P* = 0.5) [12].

Roh et al. performed a systematic review and meta-analysis, including 1,755 patients, to compare RC with LC. The study concluded that LC had a shorter operative duration (*P* < 0.05), lower overall complication rate (*P* < 0.05), and reduced operative costs (*P* < 0.05), when compared to RC. However, RC had reduced intraoperative blood loss (*P* < 0.05). Finally, the subgroup analysis showed that RC had a lower conversion rate to open surgery and a shorter duration of hospitalization compared to LC [13].

Ma et al. performed a systematic review and meta-analysis to compare RRC with LRC involving 7,769 patients. The authors concluded that LRC was associated with a longer duration of hospitalization when compared to RRC (MD -0.85; 95% CI -1.07 to -0.63; *P* < 0.00001), but with shorter operative duration (MD = 43.61; 95% CI 39.11-48.10; *P* < 0.00001). However, RRC was associated with significantly lower complication rates (OR 0.73; 95% CI 0.52-1.01; *P* = 0.05), lower blood loss (MD -16.89; 95% CI -24.80 to -8.98; *P* < 0.00001), and lower conversion rate (OR 0.34; 95% CI 0.15-0.75; *P* = 0.008), when compared to LRC. No differences were observed in bowel function recovery [14].

Park et al. performed a prospective randomized clinical trial to investigate the differences between RC and LC involving 71 patients. The trial concluded that RC required longer operative time (195 minutes vs. 129 minutes for LAC; *P* < 0.001) and was associated with higher costs, with average costs of US$12,235 versus US$10,319 for LC (*P* = 0.013). Moreover, the five-year disease-free rate and five-year survival rate were similar for both approaches: 77.4% in RC (95% CI 60.6%-92.1%) versus 83.6% in LC (95% CI 72.1%-97.0%; *P* = 0.442) and 91.1% in RC (95% CI 78.8%-100%) versus 91.0% in LC (95% CI 81.3%-100%; *P* = 0.678), respectively [15].

Waters et al. performed a systematic review to compare RRC and LRC. The study involved 4,072 patients. The study concluded that RRC had faster bowel function recovery (2.0-2.7 days) compared to LRC (2.5-4.0 days) (*P* < 0.05), lower anastomotic leakage (0% vs. 8.3%; *P* < 0.05), decreased duration of hospitalization, lower rates of incision hernias, lower rates of conversion to open surgery, higher resection rates (*P* < 0.001), and a higher number of lymph nodes harvested (*P* < 0.05). No differences were observed in the 30-day mortality rate and postoperative ileus. Finally, the study outlined a lack of patient-oriented outcomes analysis [16].

In 2021, a meta-analysis by Flynn et al. compared RC with LC. The authors concluded that the robotic approach had a lower learning curve, requiring only 16 cases to achieve proficiency, compared to 25 cases for LC. Also, it had lower overall complications (14.6% vs. 0%; *P* = 0.013) compared to laparoscopic surgery. Finally, the authors also investigated the outcomes in simulation studies and concluded that RC had lower operative duration as well as lower error rates when compared with the laparoscopic approach [17].

A systematic review and meta-analysis by Zhu et al. compared RRC with LRC while involving 1,180 patients in the analysis. The study concluded that RRC was associated with a greater number of harvested lymph nodes (weighted MD [WMD] 1.47; *P* = 0.05), reduced blood loss (WMD -13.43; *P* = 0.0003), lower rate of conversion to open surgery (OR 0.30; *P* < 0.0001), but with a higher operative duration (WMD 65.20; *P* < 0.00001) when compared to LRC. No major differences were observed between the two approaches regarding bowel function recovery, length of hospitalization, reoperation rates, complication rates, mortality rates, wound infection, and anastomotic leakage [18].

Another systematic review and meta-analysis performed by Genova et al. compared RRC with LRC. It included 24,193 patients. The authors observed that RRC was associated with a shorter duration of hospitalization, decreased blood loss, reduced conversion to open surgery, quicker bowel function recovery, and lower overall complication rates compared to LRC. However, RRC was associated with longer operative time and with higher costs [19].

Tschann et al. performed a systematic review and meta-analysis to compare RRC with LRC. The study involved 30,356 patients. The authors observed that RRC required more operative time (MD -42.01 minutes LRC vs. RRC; *P* < 0.001) but was associated with reduced blood loss (MD 10.03; *P* = 0.02), lower conversion rate to open surgery (OR 1.65; *P* < 0.001), and shorter duration of hospitalization (MD 0.84 days; *P *= 0.003) compared to LRC. Finally, no major differences were observed between the two approaches regarding long-term oncological outcomes [20].

A systematic review and meta-analysis by Zheng et al., which included 5,152 patients, compared RRC with LRC. The study concluded that RRC had lower conversion rates (*P* = 0.03), shorter duration of hospitalization (*P* = 0.01), but longer operative time (*P* < 0.001), compared to LRC. Moreover, comparable outcomes were observed between the two techniques regarding blood loss, lymph node retrieval, and overall complications [21].

A systematic review and meta-analysis by Zheng et al., which included 15,241 patients, compared RRC with LRC. The study showed that RRC had a shorter duration of hospitalization, lower conversion rate, faster bowel function recovery, lower rate of overall complication, and higher number of harvested lymph nodes compared to LRC. However, it was associated with longer operative time and higher costs [22].

A systematic review and meta-analysis by Kim et al. involved 523 patients and compared RRC with LRC. The study concluded that the number of harvested lymph nodes, disease-free survival, and overall survival did not differ between the two approaches [23].

Discussion

Strengths and Limitations

The literature review comprises several different studies comparing RRC and LRC in patients with colorectal cancer. Nonetheless, the comprehensive review comes with both strengths and limitations. A notable strength of the review is its inclusion of both meta-analyses and/or systematic reviews, along with randomized clinical trials, which represent some of the highest levels of evidence. Additionally, certain studies, such as Chang et al. [9] and Duan et al. [11], include a significant number of patients. The substantial enrollment of patients in these analyses further enhances the reliability and validity of the conclusions. In addition, some studies have assessed several different outcomes, such as operative duration, blood loss, length of hospitalization, long-term survival, and hospitalization rates. For example, Tschann et al. not only reported results from the operative perspective but also examined oncological outcomes [20]. Such studies provide a complete and holistic view of the surgical approaches and guide surgeons and patients. On the other hand, this review has its limitations. The current literature lacks randomized clinical trials, necessitating the inclusion of meta-analyses and systematic reviews to offer a more comprehensive view of the outcomes of surgical techniques. While incorporating different types of studies introduces variation, it also allows for potential inconsistencies in data interpretation. This is exemplified by the data on operative time, where certain studies tend to disagree. Another limitation is the limited focus on cost and surgical efficiency in the reviewed studies. While works such as those by Park et al. [7] and Solaini et al. [12] discuss economic implications, there is generally more emphasis on the operative aspect rather than the holistic implications that the approach may have on the patient, including the economic burden. Finally, some studies favor one approach over the other. Even though there is a clear trend, more focus should be placed on whether an advantage of an approach truly translates into better patient-centered outcomes. For instance, does having slightly lower intraoperative blood loss via the robotic approach genuinely improve patient-centered outcomes? This inconsistency needs to be addressed in future research.

Research gaps and future directions

This comprehensive literature review includes various studies comparing RRC and LRC approaches for colorectal cancer. It provides a detailed insight into these surgical techniques, outlining their pros and cons. The future direction of research in this area could be multi-faceted, aiming to address the research gaps and build on the strengths identified in these studies.

First, there is a need for more randomized clinical trials with larger sample sizes, similar to the study conducted by Park et al. [7]. These trials should attempt to provide more definitive conclusions about the safety and efficacy of RRC compared to LRC. Additionally, future studies should consider including a broader range of patient demographics to enhance the generalizability of the findings and be more valid for populations outside the demographic target of the particular hospital. Multicenter randomized clinical trials could further assist in this attempt.

Furthermore, given the variance in some outcomes, like operative duration and costs, observed in studies like those by Xu et al. [7] and Park et al. [8], future research should focus on optimizing robotic surgery techniques to reduce operative times and costs. This could involve the development of new surgical tools, the incorporation of existing tools, such as artificial intelligence or machine learning, or the refinement of existing robotic systems. This could potentially improve surgical precision, reduce complications, and enhance patient outcomes.

Studies such as those by Chang et al. [9] and Solaini et al. [12] highlight the importance of examining long-term outcomes, such as patient quality of life post-surgery. Future studies should continue to assess these outcomes to better understand the long-term benefits and potential drawbacks of each surgical method and to an extent allow patients and physicians to make informed choices whenever choosing an approach.

Surgical site infections (SSIs) are also a common problem. An observational study by Panos et al. found that patients over 70 years old, with a BMI of 30 kg/m² or higher, diabetes, chronic steroid therapy, an ASA score of 2 or higher, or those who underwent open surgery were more prone to SSIs, particularly with Escherichia coli and enterococcus species. This should be taken into account by healthcare providers to identify high-risk patients and provide preventative measures against SSIs [24].

The reviewed research indicates a general trend toward the effectiveness of robotic surgery in certain aspects, such as lower complication rates and quicker recovery times. However, these benefits often come with higher costs and longer operative times. Thus, future research should focus on cost-benefit analyses to help patients and physicians make more informed decisions.

Potential biases

A major bias that cannot be ignored is the selection bias, particularly in the observational studies included in the systematic reviews. For example, the choice of a surgical method could have been influenced by both patient factors and surgeons’ experience. Moreover, there could be a presence of publication bias, as studies with positive results are more likely to be published. This overrepresentation of positive findings could skew the results and extend their interpretation in favor of one of the approaches over the other. Also, focusing extensively on the surgical outcomes of the approaches can overshadow patient-reported outcomes that are underrepresented in the literature. Finally, technological advancements have occurred in the last decade, particularly in the field of robotic surgery. To some extent, earlier studies may have become less relevant to current practice.

Impact on results and interpretations

The consistently longer operative times reported for RRC across several studies might reflect a learning curve that has not plateaued yet or the inherently complex nature of the technology, a factor not applicable to laparoscopy. This needs to be balanced against potential benefits such as precision and reduced blood loss, with surgeons' experiences taken into account in future studies. Furthermore, major findings in some studies include lower complication rates and faster recovery in RRC. However, the clinical relevance of these differences needs to be further explored, especially in the context of long-term patient outcomes. In addition, the higher costs associated with robotic surgeries are a consistent finding. Future research must delve into cost-benefit analyses, considering long-term outcomes and patient satisfaction. Additionally, an analysis is required to determine whether the expenses are associated with the initial high-value purchase of the robotic system or with day-to-day usage and maintenance. Furthermore, the large and diverse patient populations in these studies enhance the generalizability of the results. However, individual patient factors and specific clinical scenarios may necessitate a tailored approach. Further subgroup analyses may be required to determine specific approaches for specific patient populations (i.e., cancer location and stage). Concerning long-term outcomes, studies such as the one by Kim et al. [23], which focuses on long-term outcomes, are crucial. They provide insights beyond the immediate postoperative period, which is essential for patient-centered care, especially when examining an approach practiced in thousands of patients per year.

Implications for clinical practice

The choice of surgical approach depends on well-weighted and informed decisions from both the physician and the patient. The studies indicate that both RRC and LRC have their unique advantages and drawbacks. For instance, RRC often results in lower blood loss and faster recovery but entails longer operative times and higher costs. This knowledge should guide surgeons in making informed decisions based on specific patient needs while taking into account hospital resources. Moreover, given the variability in operative duration and complication rates, surgical training programs need to improve and adapt in the modern and fast-changing surgical field. The learning curves for robotic surgeries, as highlighted in some studies, suggest the need for focused training in robotic techniques. This will assist young surgeons, especially, in reaching proficiency in a faster and more resourceful way. Finally, clinicians should inform patients about the potential benefits and limitations of each surgical approach. This includes discussing operative times, recovery expectations, and total costs.

Implications for policymaking

The higher costs that are associated with RRC necessitate policy interventions. Health insurance providers and hospitals should consider strategies to make these technologies more accessible and cost-effective, especially in less developed countries. Thus, institutions should invest in the research and development of robotic technologies, and policymakers should evaluate the long-term benefits of investing in such technologies while simultaneously balancing the immediate costs and potential improvement in patient outcomes. Finally, there is a need for standardizing surgical protocols across researchers. Different protocols will yield different results and, to some extent, may introduce biases.

## Conclusions

RRC provides patients with lower complication rates and quicker recovery times but comes at the expense of longer operative duration and higher costs compared to LRC. Variability is noted in the results due to the inclusion of different types of studies in the review. This necessitates further multicenter randomized clinical trials, which focus on safety, efficacy, long-term patient quality of life, and the cost-benefit aspects of each approach. Future directions would aim to make robotic surgery more efficient and cost-effective, address potential biases explored in current literature, and re-evaluate older studies against recent technological advances. Based on the current evidence, the decision between RRC and LRC should be based on patient characteristics and hospital resources. Emphasis should also be placed on surgical programs to bring surgeons up to speed with recent advances and help them navigate the learning curve of robotic surgery. Finally, there is a great need to standardize research protocols to reduce biases and inconsistencies in research.
